# Slinker: Visualising novel splicing events in RNA-Seq data

**DOI:** 10.12688/f1000research.74836.1

**Published:** 2021-12-07

**Authors:** Breon Schmidt, Marek Cmero, Paul Ekert, Nadia Davidson, Alicia Oshlack

**Affiliations:** 1 Peter MacCallum Cancer Centre, Melbourne, Victoria, 3000, Australia; 2 School of BioScience, University of Melbourne, Melbourne, Victoria, 3000, Australia; 3 Sir Peter MacCallum Department of Oncology, University of Melbourne, Melbourne, Victoria, 3000, Australia; 4 Walter and Eliza Hall Institute of Medical Research, Parkville, Victoria, 3052, Australia; 5 Murdoch Children's Research Institute, Parkville, Victoria, 3052, Australia; 6 Children’s Cancer Institute, Lowy Cancer Centre, Kensington, New South Wales, 2033, Australia; 7 School of Women’s and Children’s Health, University of New South Wales, Sydney, Victoria, 2052, Australia

**Keywords:** RNA-Seq, Visualisation, Novel Splicing Events, superTranscripts, bioinformatics

## Abstract

Visualisation of the transcriptome relative to a reference genome is fraught with sparsity. This is due to RNA sequencing (RNA-Seq) reads being predominantly mapped to exons that account for just under 3% of the human genome. Recently, we have used exon-only references, superTranscripts, to improve visualisation of aligned RNA-Seq data through the omission of supposedly unexpressed regions such as introns. However, variation within these regions can lead to novel splicing events that may drive a pathogenic phenotype. In these cases, the loss of information in only retaining annotated exons presents significant drawbacks. Here we present Slinker, a bioinformatics pipeline written in Python and Bpipe that uses a data-driven approach to assemble sample-specific superTranscripts. At its core, Slinker uses Stringtie2 to assemble transcripts with any sequence across any gene. This assembly is merged with reference transcripts, converted to a superTranscript, of which rich visualisations are made through Plotly with associated annotation and coverage information. Slinker was validated on five novel splicing events of rare disease samples from a cohort of primary muscular disorders. In addition, Slinker was shown to be effective in visualising deletion events within transcriptomes of tumour samples in the important leukemia gene, IKZF1. Slinker offers a succinct visualisation of RNA-Seq alignments across typically sparse regions and is freely available on Github.

## Introduction

Genomic variants often carry through to the transcriptome. Through these events, gene products can be inhibited, upregulated, or modified, which disrupts typical function. Understanding how a genomic variant has altered transcription can offer insights into causal mechanisms for pathogenesis or potential targets for therapeutic intervention. ^1^^,^^2^ DNA variants can be identified through genome sequencing and transcriptional disruption can be measured by RNA sequencing (RNA-Seq). In addition, RNA-Seq data can be used to detect an assortment of variants such as single nucleotide polymorphisms (SNPs), deletions or insertions, and altered splicing. ^2^^–^^6^ It is common practice to visualise any novel events relative to control samples with tools such as the Integrative Genomics Viewer (IGV). ^7^ This process involves aligning sequencing reads to a reference genome. However, when visualising RNA-Seq data, the size of introns are typically significantly larger than the exons that harbour most of the aligned reads. ^8^^,^^9^ As a consequence, these visualisations are sparse. The intronic regions with low coverage offer little information, yet significantly reduce the interpretability of the transcribed regions.

In an effort to provide succinct representations of RNA-Seq data, we previously developed the concept of superTranscripts. ^10^ A superTranscript is the flattened collection of transcripts within a gene and includes only the concatenated sequence of its exons. Subsequently, alignment to a superTranscript reference mostly resolves the sparsity problem through elimination of low-information intronic regions. This concept was further extended in our previous work with the release of Clinker, which uses superTranscripts to visualise fusion genes. ^11^ In the Clinker pipeline, the pre-generated superTranscripts of genes identified as belonging to a fusion are combined to form a new reference. The sequencing reads are then mapped to this with the splice-aware alignment tool, STAR. ^12^ Any splice junctions spanning the two genes in the superTranscripts are indicative of a fusion breakpoint. Although this method offers succinct representations of fusion events, it is not applicable to other types of events that can produce novel structures in transcripts. Specifically, in both the rare disease and cancer context, it is important to visualise any events that might occur within the excised intronic regions, or those that add sequences to the reference transcripts upstream or downstream of annotated exons.

**Figure 1.**
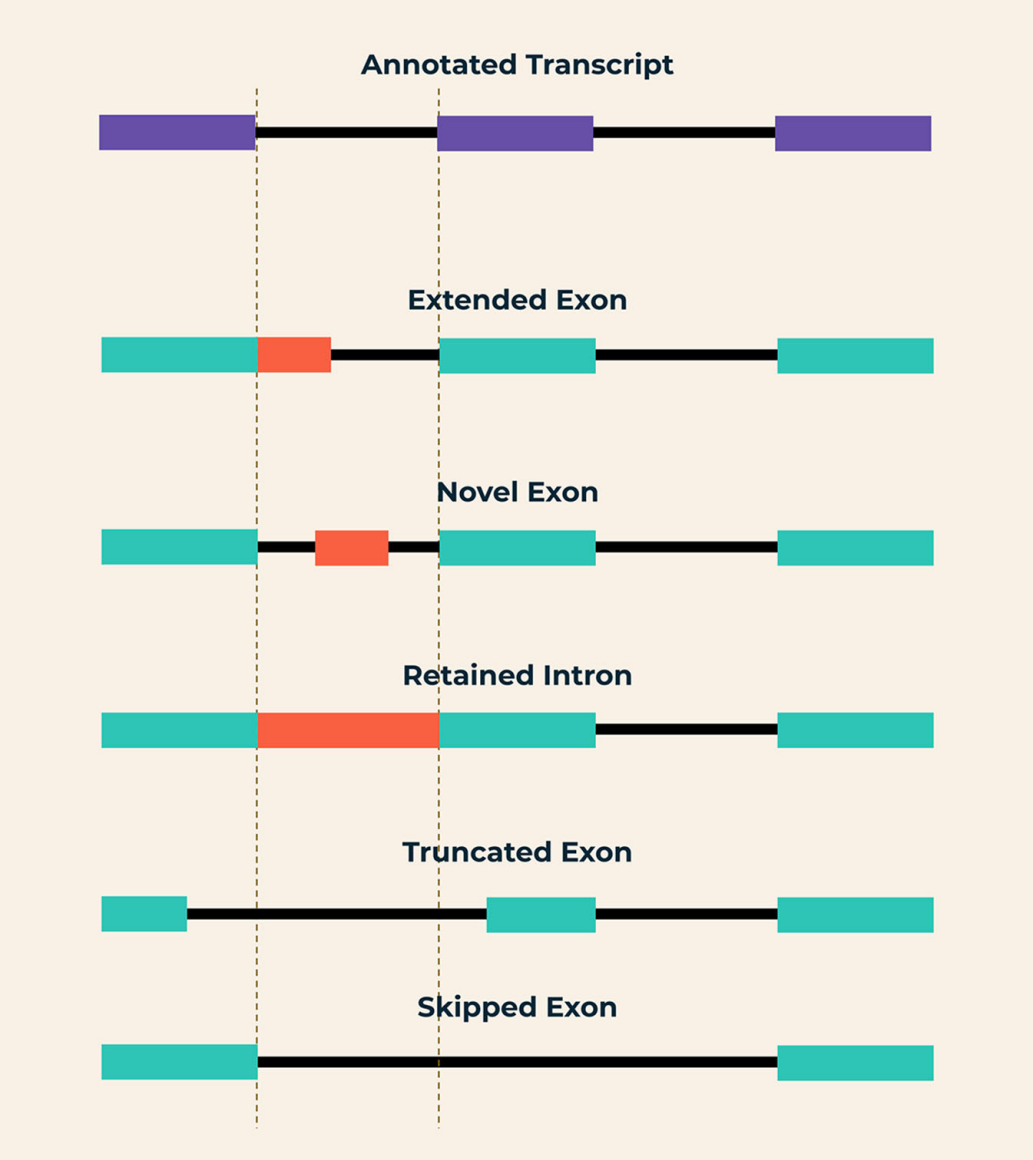
Depiction of each of the novel splicing events that Slinker is designed to visualise. Compared to the reference annotation: retained introns are defined by the inclusion of regions between exons, up to and including the exon boundary. Novel exons are defined by the inclusion of regions between exons, not including the exon boundaries. Skipped exons are defined by the existence of a splice junction in the case that starts and ends at known exon boundaries, but does not exist within the controls. Truncated exons occur when an exon in the case does not extend to the annotated exon boundary. Conversely, an extended exon is where an assembled exon within the case exceeds an annotated exon boundary.

To address these requirements in the visualisation of novel transcripts, we present Slinker, a superTranscript generation and visualisation method. Slinker builds on the concept of visualisation using superTranscripts, but uses genome-guided assembly rather than predefined annotation to incorporate the novel transcribed regions of interest into a reference. Through this, Slinker retains novel transcribed regions outside of annotated exons, that can be described as: novel exons, retained introns, skipped exons, truncated exons, and extended exons ( Figure 1).

These events can be an important consequence of genomic variants and may be associated with a disease phenotype. To demonstrate the utility of Slinker, we applied it to rare disease variants, discovered with the aid of RNA-Seq in genes associated with primary muscle disorders in skeletal muscle ^2^ and events detected in childhood leukemia. ^6^^,^^13^ We determined that Slinker offers a succinct and complementary method to visualise and explore RNA-Seq data. Slinker is a Bpipe pipeline publicly available via Github ( *Extended data*).

## Methods

**Figure 2.**
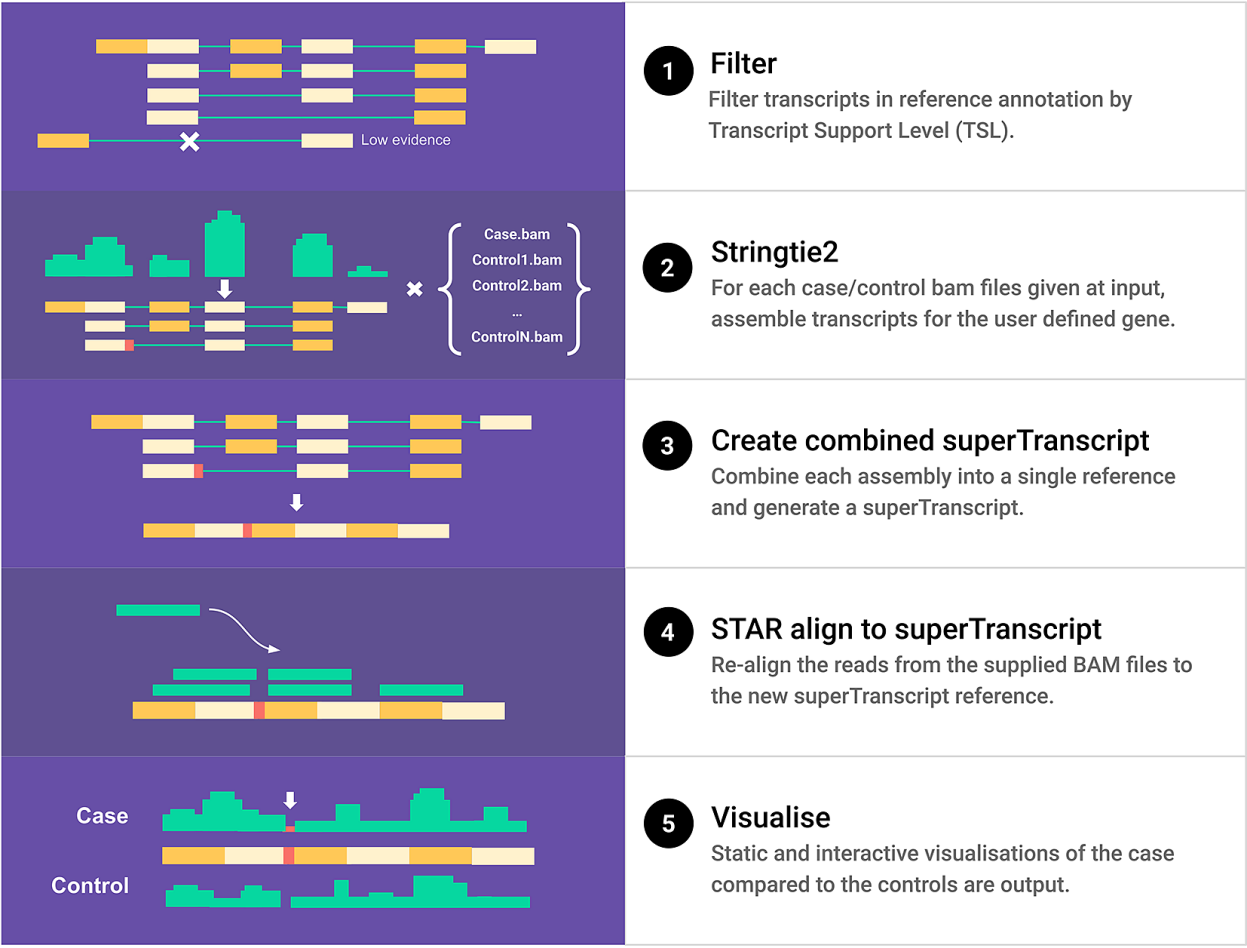
A schematic of the Slinker pipeline for a single user-input gene. Reference transcripts are first filtered by Transcript Support Level (TSL) as indicated in the reference annotation. The TSL is a tag included within Ensembl annotation that informs on the size and quality of evidence supporting the existence of the associated transcript. These transcripts, along with the input BAM files, are used to perform genome-guided assembly and output the predicted transcripts for each control and the case sample. The merged result of these assemblies is then flattened, exons concatenated, and oriented in the transcriptionally forwards direction to create the genome-guided superTranscripts. The reads previously mapped to the user-supplied gene are extracted from each input BAM and then re-aligned to this new reference. Finally, the coverage from each alignment, splice junctions, and transcript annotation are combined into both interactive and static visualisations upon completion of the pipeline.

Slinker is a pipeline that is used to visualise novel gene events using RNA-Seq. The main steps in the pipeline are outlined in Figure 2. Slinker requires multiple inputs: a gene name, aligned RNA-Seq reads (for the case and each control), a genomic sequence reference, and a transcriptome reference annotation. A custom annotation can also be supplied. These must be provided in GTF format with the naming conventions for the attributes reflecting those used within Ensembl annotations. ^14^ Upon completion, a PNG file containing a static image of the visualisation and an interactive HTML version are generated and saved to a user-defined destination.

The first step in the Slinker pipeline is to filter the gene transcripts to be visualised by the level of support in the Ensembl annotation. Specifically, all transcripts that either have a Transcript Support Level of 1 or are lacking this information are retained, the rest are filtered out. This ensures that only the best-supported transcripts are included in the assembly. The next step uses Samtools (v1.13) to extract all the reads aligned to the genomic region that the user-supplied gene defines in each input BAM. From each of these reduced BAM files, Stringtie2 (v2.1.7) performs genome-guided assembly using either the supplied annotation or the hg19 or hg38 references packaged with Slinker. ^15^ The output of Stringtie2 is a GTF file for each BAM that contains all potential reference and novel transcripts existing within that sample. The transcripts are further filtered by the Stringtie2 coverage estimate, removing any novel transcripts that do not meet the defined threshold (default, c = 1). These transcripts are then merged into a single GTF file. The reason for generating transcripts for both the case and controls is so that deletion events in the case are exposed. Next, we flatten these transcripts into a single linear representation, with concatenated exons, and then visually orient the strand left-to-right, creating a data-driven superTranscript.

A reference is then created from the generated superTranscript through gffread (v0.9.9) and STAR’s (v2.7.3a) genomeGenerate mode. ^12^^,^^16^ The reads extracted from each input BAM that overlap the gene of interest are then aligned to this new reference using STAR in alignReads mode, specifically with all splice junction score penalties set to 0. Assembled transcripts are then annotated according to the new superTranscript coordinates. Finally, a custom plotting package using the Python library, Plotly (v4.0 and above), is used to generate both a static image and interactive HTML output. These plots contain coverage and splice junction tracks for the test sample and controls with the superTranscript annotations included for context. Slinker automatically detects and highlights novel splicing events in the gene of interest. These events are discovered through comparisons between the reference and novel transcripts and highlighted according to the novel event’s proximity to known exon boundaries, or whether an exon has been skipped. For instance, retained introns are defined by the inclusion of the region between exons in a gene, including the exon boundary. Novel exons are defined by the inclusion of regions between exons, not including the exon boundaries. Skipped exons are defined by the existence of a splice junction in the studied case that starts and ends at known exon boundaries, but does not exist within the controls. Truncated exons occur when an exon does not extend to the annotated exon boundary in the case. Conversely, an extended exon is where an assembled exon within the case exceeds an annotated exon boundary.

Slinker can be executed through a single command line option, where the inputs and outputs of each particular stage of the pipeline are handled by Bpipe. ^17^ However, users who do not wish to use Bpipe may also run each step manually. The core dependencies of Slinker are the STAR aligner, Stringtie2, Plotly (v4 and above), gffread, and Samtools. ^12^^,^^15^^,^^16^^,^^18^^,^^19^ Runtime for a single gene is under 10 minutes with 20gb of memory and four cores allocated, with a control and case sample containing approximately 125 and 275 million reads, respectively.

## Results

In order to demonstrate the utility of Slinker for visualising transcriptional variants that are associated with rare disease, we applied it to muscle biopsy samples obtained from Cummings *et al.* ^2^ Of these, four were selected for visualisation based on the existence of relevant novel splicing events in disease related genes ( Table 1). These events were found to have consensus between Cummings *et al.* ^2^ and the variant caller, MINTIE. ^6^ Three skeletal muscle controls were selected from the Genotype-Tissue Expression (GTEx) consortium that were also used as controls within Cummings *et al.* ^2^^,^^20^ The selected samples were then aligned to hg38 with the STAR aligner in 2-pass mode, of which the resulting BAM files were inputted into the Slinker pipeline. ^12^

**Table 1.** A list of the cases and controls used within this study to validate Slinker. The GTEX samples were used as controls for the COL6A1, POMGNT1, RYR1, and NEB genes. The B-ALL20_7 and B-ALL9_4 samples were validated through Multiplex-ligation dependent probe amplification (MLPA) to not contain IKZF1 deletions and were used as controls against samples that were validated to have DEL4-7, DEL2-7, and DEL4-8.

Sample ID	Gene	Variant	Source	Verified
Cases				
SRR5020918	COL6A1	Novel Exon	( Cummings *et al.* 2017)	MINTIE
SRR5034830	POMGNT1	Skipped Exon	( Cummings et al. 2017)	MINTIE
		Retained Intron	( Cummings *et al.* 2017)	MINTIE
SRR5033001	RYR1	Truncated Exon	( Cummings *et al.* 2017)	MINTIE
SRR5038795	NEB	Extended Exon	( Cummings *et al.* 2017)	MINTIE
B-ALL18_4	IKZF1	DEL4-7	( Brown *et al.* 2020)	MLPA
B-ALL19_11	IKZF1	DEL2-7	( Brown *et al.* 2020)	MLPA
B-ALL7_8	IKZF1	DEL4-8	( Brown *et al.* 2020)	MLPA
Controls				
SRR811771	Multiple	N/A	( Carithers *et al.* 2015)	N/A
SRR810249	Multiple	N/A	( Carithers *et al.* 2015)	N/A
SRR809595	Multiple	N/A	( Carithers *et al.* 2015)	N/A
B-ALL20_7	IKZF1	N/A	( Brown *et al.* 2020)	MLPA
B-ALL9_4	IKZF1	N/A	( Brown *et al.* 2020)	MLPA

**Figure 3.**
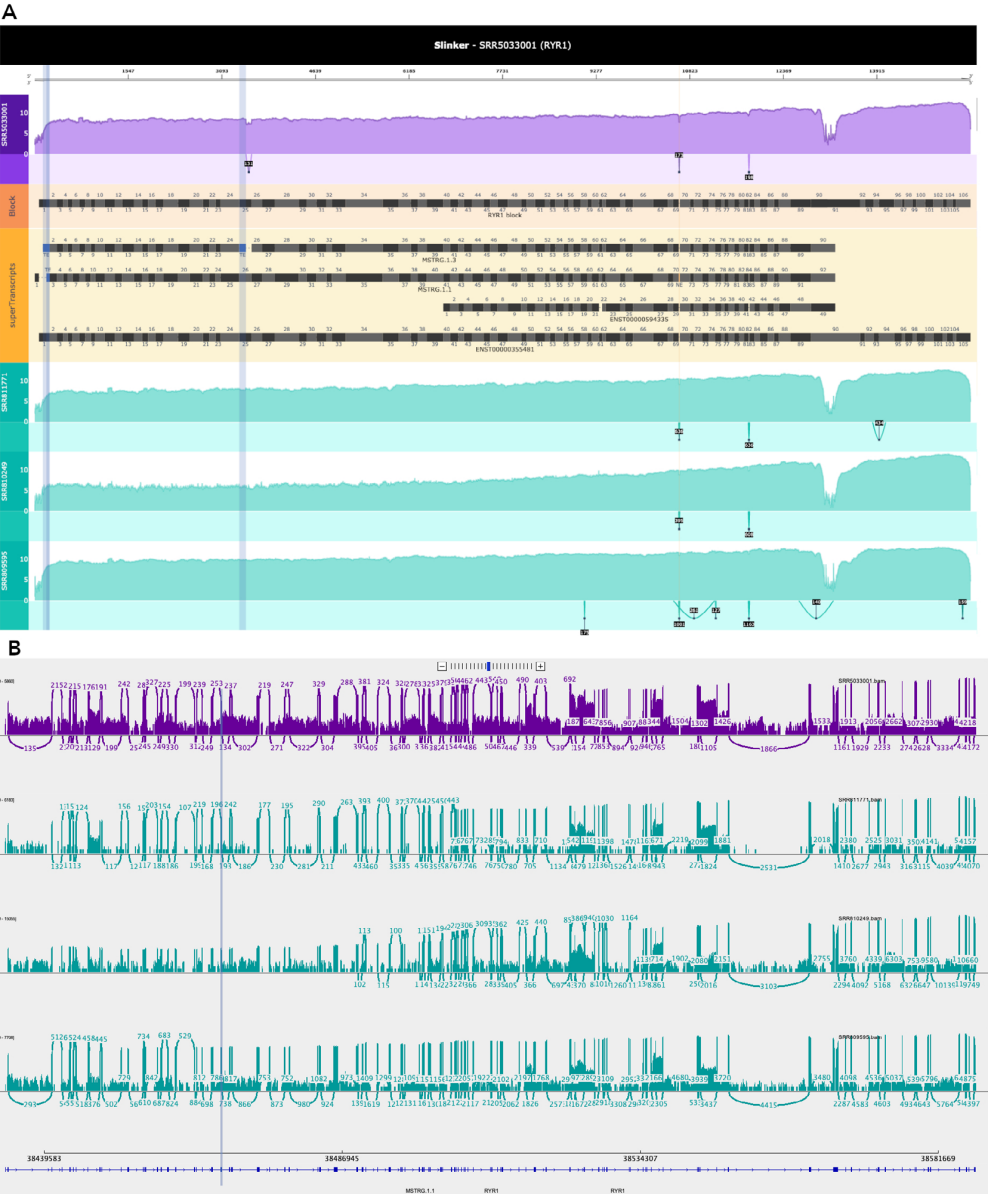
Integrated Genomics Viewer (IGV) and Slinker visualisations of the RYR1 gene. A. Slinker visualisations can show coverage (log scale) across the entire gene including events such as truncated exons (purple shading with adjacent drop in coverage), in a single, compact visualisation. Such a visualisation is desirable as users can consider the event in the greater context of the gene's expression. B. Integrative Genomics Viewer (IGV) Sashimi plot including the same case, controls, and transcripts as in A but not in the superTranscript format. Case is shown in purple and the controls in teal. The truncated exon is highlighted with the purple vertical line.

Figure 3 demonstrates Slinker’s superTranscript visualisation for the RYR1 gene, which has 105 exons across 154kb of the genome. This was compared to the visualisation of the same region using a refined IGV sashimi plot. Log coverage settings were enabled in both Slinker and IGV. Slinker clearly revealed the novel event of a truncated exon in exon 25, which was far less obvious in the genomic view that included the unnecessary splice junctions and alignment sparsity.

**Figure 4.**
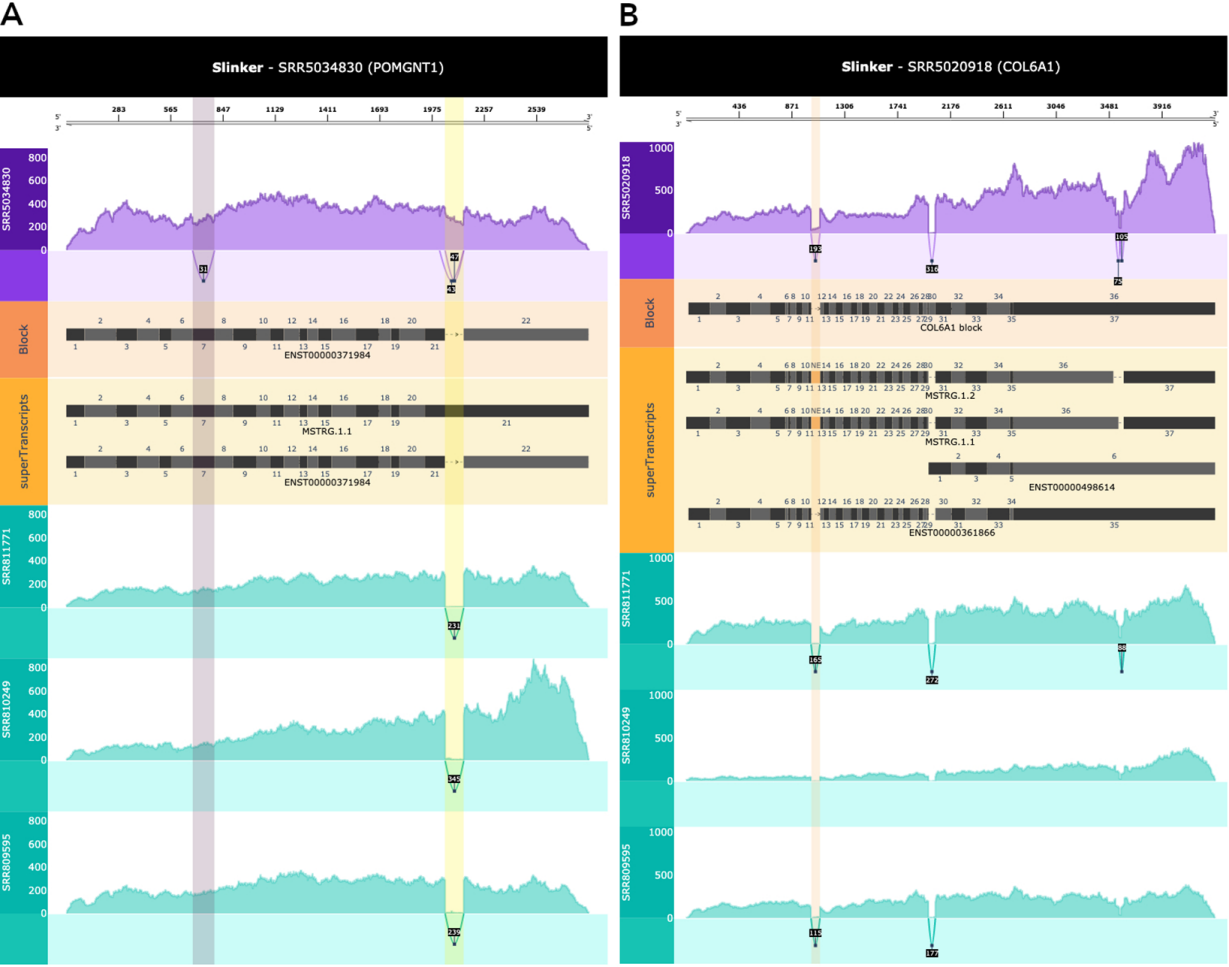
A. Slinker visualisation of the POMGNT1 gene in a sample containing two variants which resulted in both removed and added sequence. A skipped exon (purple vertical highlight) and a retained intron (yellow vertical highlight) are highlighted. From the top: Title bar, Axis in superTranscript coordinates, case’s coverage in raw counts, case’s splice junctions, superTranscript of only the reference transcripts, novel and reference superTranscripts as defined by Stringtie2, and a coverage and junctions track for each control. Slinker highlights relevant novel splicing events (retained introns, novel exons, extended exons, truncated exons, and skipped exons) in a distinct colour to enable users to quickly identify what variation exists between a case and controls. B. Slinker visualisation of COL6A1 in a sample with a novel exon event (orange vertical highlight) between exons 11-12.

**Figure 5.**
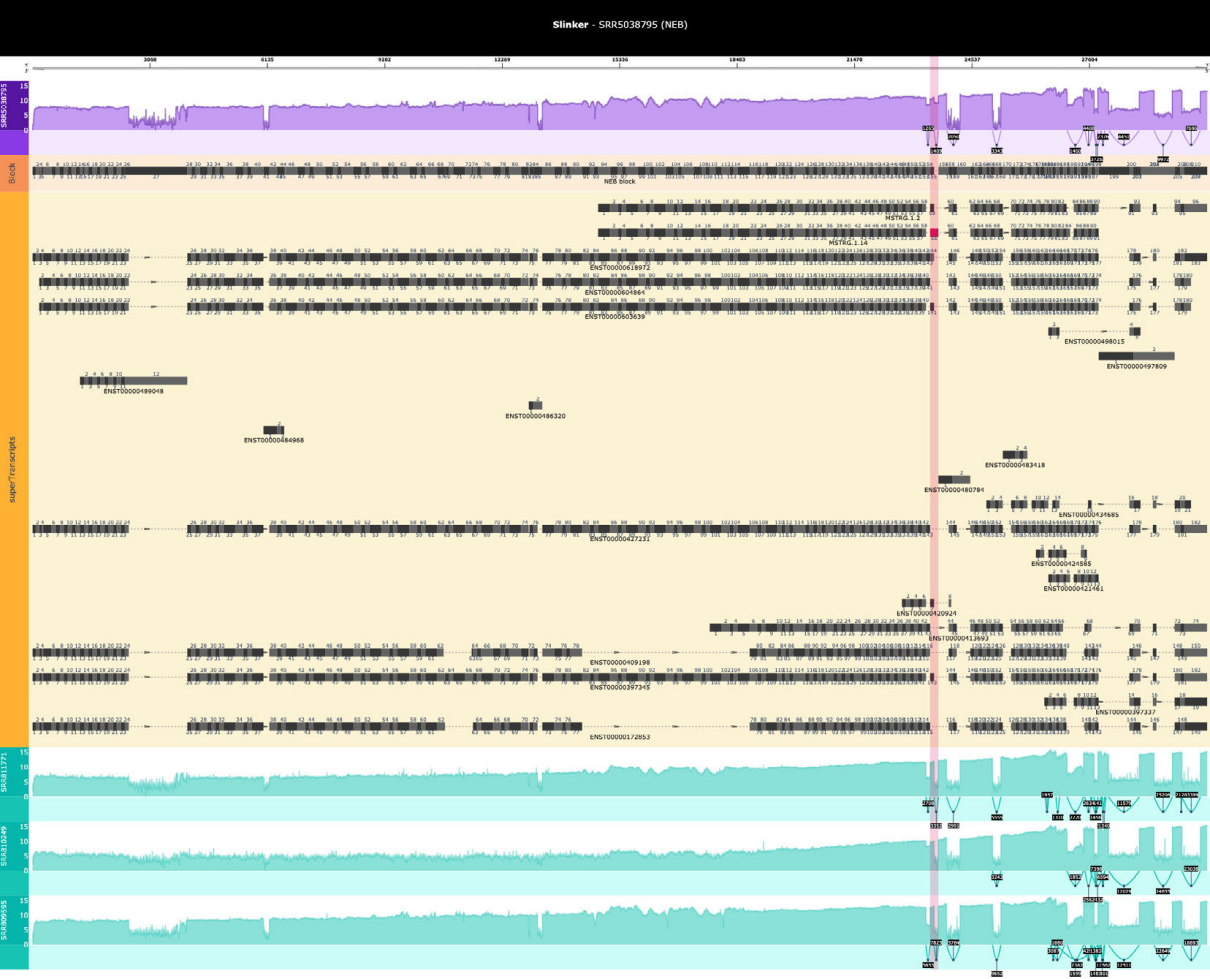
Slinker visualisation in a sample with an extended exon event (red vertical highlight) between exons 143-144 in the NEB gene. In this case, the TSL filter was not applied and a reference with all transcripts was passed through to Slinker. This was due to the large number of transcripts of NEB, which were assembled by Stringtie and masked the interesting event. This demonstrates the utility of being able to pass through custom annotations which may be more suitable to the visualisation at hand.

Next, we demonstrated Slinker’s ability to visualise a range of novel events involving both added and deleted sequences in transcripts, each confirmed through MINTIE. Figure 4A depicts two novel splicing events in POMGNT1 found within a single case sample, and is highlighted through a comparison with the three GTEX controls ( Table 1). In this case, we could see a retained intron in the genome-guided assembly (highlighted in yellow) and an exon-skipping event (highlighted in purple) in exon 7. Applying Slinker to the COL6A1 gene in another sample clearly revealed the novel exon as reported by Cummings *et al.* ^2^ ( Figure 4B). Furthermore, a truncated exon is shown in the RYR1 gene ( Figure 3A) and an extended exon is shown within the NEB gene ( Figure 5).

**Figure 6.**
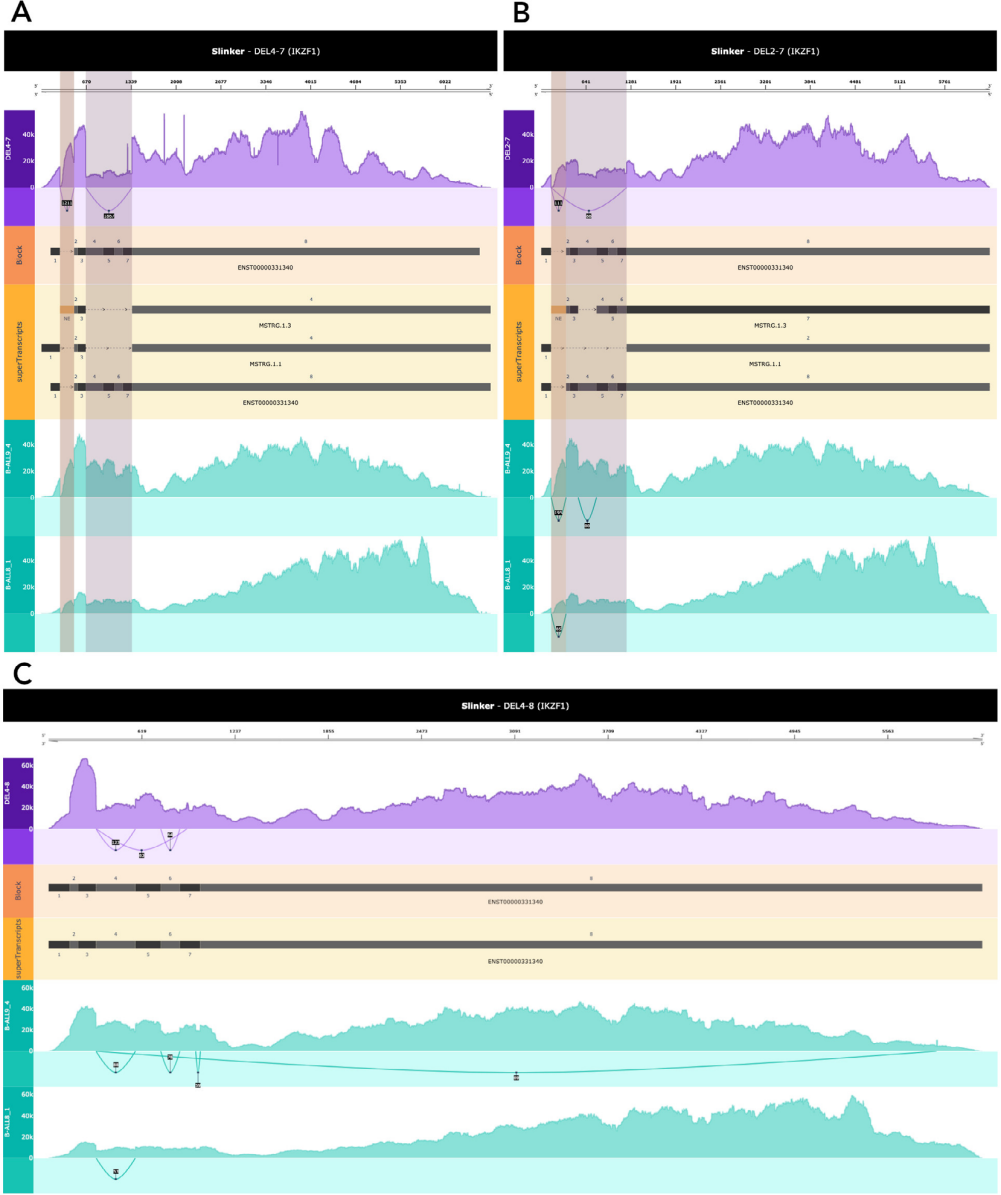
Deletions within the IKZF1 gene. A. An exon 4-7 deletion in IKZF1. A clear dip in coverage in the case compared to the controls can be seen within the purple highlighted region. Slinker was run using only a single transcript (ENST00000331349), which was representative of the canonical. The highlighted novel exon was annotated in a transcript that is within the greater reference. Transcripts were filtered using the minimum coverage parameter to include all assemblies with the known deletion event. Coverage was normalised by total reads in IKZF1. B. A 2-7 deletion in IKZF1. A clear dip in coverage in the case in comparison to the controls can be seen within the purple highlighted region. In this figure, Slinker was run using only a single transcript (ENST00000331349) which was representative of the canonical. Coverage was normalised by total reads in IKZF1. C. A 4-8 deletion in IKZF1. This comparison demonstrates a slight change in coverage post exon 3 to the end of the transcript in the case. This is representative of the deletion event known to have occurred. Coverage was normalised by total reads in IKZF1.

Finally, we applied Slinker to three childhood B-Cell Acute Lymphoblastic Leukemia samples obtained from the Royal Children’s Hospital (RCH), that harboured various IKZF1 deletions; these were validated in DNA using a multiplex-ligation-dependent probe amplification (MLPA) assay ( Table 1). Figure 6A depicts an exon 4-7 deletion within one such case compared to two other leukemia samples in the RCH cohort. These two samples were chosen due to their high IKZF1 expression relative to other samples within the cohort, but did not contain a deletion either. In addition to the automatic highlighting denoting a SE event, a clear drop in expression across exons 4 and 7 can be seen in the case relative to the control samples. The 2-7 deletion event ( Figure 6B) was also clearly highlighted as a SE event. However, this was not true for the 4-8 deletion sample as no splice junctions existed and the deletion included the final exon ( Figure 6C). However, the drop in coverage between the case and controls in this succinct form demonstrates the utility of Slinker for providing a visual comparison between any samples. An interesting aspect of the 4-7 IKZF1 deletion was the assembly of multiple transcripts containing the deletion event ( Figure 6B). This demonstrates that Slinker may help provide more information for deletion events over exclusive alignment to predefined superTranscripts.

## Discussion

DNA mutations can be causal drivers of disease. ^2^^,^^6^ While genomic sequencing is becoming more commonly used to diagnose genetic diseases, there are still many cases where the variants cannot be directly identified as disease-causing, and are therefore called variants of unknown significance. Mutations with the potential to impact splicing are of particular interest. One way to assess the effect of a variant is to investigate the resulting transcript using RNA sequencing and compare these transcripts to a set of controls. While there are several approaches proposed for this purpose, the visualisation of this data can be improved. ^2^^,^^21^^,^^22^ Here we address this issue with Slinker, which was built on the superTranscript visualisation framework. One of the fundamental advantages of the superTranscript method is the removal of the visualisation sparsity due to uninformative intronic information. However, novel splicing events can retain an intronic sequence, and it is therefore necessary to first determine which sequences are transcribed in the sample. Slinker utilizes the Stringtie2 data-driven method for assembling the transcripts in the sample which is then combined with reference transcripts to capture all potentially expressed sequences.

Due to the large insertion of sequences relative to the superTranscript, retained introns and novel exons were expected to be the simplest events to visualise with Slinker. This is reflected in Figure 4A and 4B. The same is true for exon skipping, where the existence of a splice junction at annotated exon boundaries in the case versus lack thereof in the control was clear ( Figure 4A). However, truncated and extended exons appeared to be less obvious in Slinker given the relatively small amount of sequence which were removed/added ( Figures 3A and 5). Nevertheless, the bespoke superTranscripts, in conjunction with the highlighting and comparisons with controls could reveal these types of events in the static visualisation even in the largest of genes ( Figure 3A). In addition, Slinker generates an interactive plot so that the user can simply zoom in on these regions to better understand the event whilst also zooming out to appreciate the greater context if required.

Though Slinker was developed to highlight novel splicing events in rare diseases, it can also be used to visualise cancer transcripts. Slinker was applied to three B-Cell Acute Lymphoblastic Leukemia (B-ALL) cases harbouring 4-7, 4-8, and 2-7 IKZF1 deletions. Deletions within this gene are a known risk factor in aggressive forms of this cancer and are used to monitor disease progression. ^13^ In each of these examples, a clear deletion event could be seen through either the presence of skipped exons or the relative drop in coverage when compared to the controls ( Figure 6). However, generally, cancer is a genetically complex disease and consequently a genetic visualisation is also challenging. ^23^ Nevertheless, these results demonstrate that Slinker can be an appropriate choice for providing a succinct visualisation of this complexity and may be applicable to a broader set of diseases.

Further work may involve improving this aspect of the software, including highlighting other novel events, such as inversions and duplications. However, this is limited by the assembler’s capacity to retrieve an accurate assembly, including the variant, and for a superTranscript to be an appropriate visual representation. ^6^

## Conclusion

Slinker is a bespoke superTranscript generation and visualisation tool with a demonstrated ability to succinctly present novel splicing events in RNA-Seq data. Its key advantage consists in removing redundant information without manual intervention, leaving room for more useful information, such as the expression across the entire gene.

We have produced a tool that has been validated on a number of known novel splice variants and is publicly available from Github ( https://github.com/Oshlack/Slinker).

## Data availability

### Underlying data

European Genome-Phenome Archive: The application of RNA sequencing for the diagnosis and genomic classification of pediatric acute lymphoblastic leukemia, https://identifiers.org/ega.study:EGAS00001004212 ^20^ This study contains the paediatric B-ALL samples (B-ALL18_4, B-ALL19_11, B-ALL7_8, B-ALL8_1, and B-ALL9_4).

NCBI dbGaP: Genetics of Inherited Muscle Disease, https://identifiers.org/dbgap:phs000655.v3.p1

NCBI dbGaP: Common Fund (CF) Genotype-Tissue Expression Project (GTEx), https://identifiers.org/dbgap:phs000424.v6.p1

### Extended data

Analysis code available from: https://github.com/Oshlack/Slinker

Archived code as at time of publication: https://doi.org/10.5281/zenodo.5719747

License: MIT

## Competing interests

No competing interests were disclosed.
