# Supplementary material for: Slinker: Visualising novel splicing events in RNA-Seq data
Source: F1000Res. 2021 Dec 7;10:1255. [Version 1] doi: 10.12688/f1000research.74836.1 (PMC8749905; doi:10.12688/f1000research.74836.1)
Supplement: Peer review history

# Peer review: Reviewer response for version 1

**DOI:** 10.5256/f1000research.78631.r118438

**Published:** 2022-02-03

**Authors:** Amy Huei-Yi Lee

**Affiliations:** 1 Simon Fraser University, Burnaby, BC, Canada

Visualizing RNA-Seq data using the reference genome via a tool like Integrative Genomics Viewer is challenged by the sparsity of transcriptome data, as RNA-Seq reads predominately map to exons. This impedes our abilities to detect SNPs, deletions/insertions and altered splicing events using RNA-Seq reads, and the associative impact on expression levels of the impacted gene. Schmidt *et al.* extend the concept of superTranscripts, which is a concatenated sequence of all the exons with a gene, with genome-guided assembly to allow visualization of novel exons, retained introns, skipped exons, truncated exons and extended exons. The manuscript is very well written with clear, detailed figures (especially the beautiful schematic of the workflow as shown in Figure 2). This is definitely a tool that I am looking forward to using in my own work.

 I only have two minor comments:

1. The colors used in Fig 1 need to be explained in the legends – and the green exons/red introns should probably be different colors to make it colorblind friendly.
2. The comparison of case and control requires coverage/sequencing depth to help with detection of some of the novel splice events (as shown in Fig 3 – 6). What happens when the sequencing depth between case and control are drastically different?

Is the rationale for developing the new method (or application) clearly explained?

Yes

Is the description of the method technically sound?

Yes

Are the conclusions about the method and its performance adequately supported by the findings presented in the article?

Yes

If any results are presented, are all the source data underlying the results available to ensure full reproducibility?

Yes

Are sufficient details provided to allow replication of the method development and its use by others?

Yes

Reviewer Expertise:

Systems immunology, multi-omics integration, neonatal sepsis, host-pathogen interactions

I confirm that I have read this submission and believe that I have an appropriate level of expertise to confirm that it is of an acceptable scientific standard.

# Peer review: Reviewer response for version 1

**DOI:** 10.5256/f1000research.78631.r102214

**Published:** 2022-01-07

**Authors:** Stephen Montgomery

**Affiliations:** 1 Department of Pathology and Department of Genetics, Stanford University School of Medicine, Stanford, CA, USA

Schmidt et al provide a pipeline for visualizing novel splicing events in RNA-seq data. The key limitations with existing approaches that they have identified are that sparsity of mapped RNA-seq reads to the genome making visualization of novel events harder to detect and further, that existing approaches are mainly guided by existing/known gene annotations. The combined approach appears useful however there are a number of minor weaknesses.

1. In practice, novel products/events are first found statistically and then visualized. It appears that the authors are suggesting that this is mostly done first by manual inspection and Slinker makes it easier to find them. From the examples provided with Slinker or with IGV it would be very difficult to eyeball the differences without considerable experience. In fact, even the authors in the figures have to highlight the critical regions so the readers know what to look at. I am not entirely sure the problem they are describing is that large or they have sufficiently solved it.
2. Overall, I think the clearly described pipeline in combination with the visualizations provides more utility and impact. I would be interested in knowing how dependent the pipeline is on the specific tools or is it compatible with specific conventional file formats and the user can change the underlying components as desired. Can the genome build be easily changed?
3. I think Figure 1 would benefit from an example of the corresponding superTranscripts. It wasn't immediately clear to me how a Skipped Exon would be conveniently visualized compared to the retained intron in Figure 2. Overall, some better documentation on how to visualize specific events as the practical examples were hard to read and very small. 
4. The examples (Fig 3, 4, 5) have abundant text that is nearly unreadable. I am a bit concerned that the output of Slinker is hard to convert into publication quality figures. Does it output as vector graphics for Illustrator/Inkscape if someone was making a multi-panel figure?
5. In some of the figures there appear to be other events but they are not highlighted. It would be interesting for the authors to comment on that. For instance in Figure 3 exon 69-70.
6. The number of controls compared to is pretty small. In practice, it may be useful to compare to 100s or 1000s of controls to find an aberrant event. Is this possible? Figure 4 focused on 3 GTEx control samples only.
7. With the splice junction penalty score set to 0, are there many more false positives than true events? How many events would visually look interesting in other randomly selected comparisons of a couple samples between genes? Does setting this score influence the recovery of the known disease event examples?
8. In the discussion, 1st para, the word mutation and variant is used interchangeably. I would recommend being more precise here.
9. You don't have to cite it. But you might find our splicePlot tool interesting too. It was designed for sQTL but one can summarize data across lots of samples. However, not with the flexibility you have here since it only focused on junctions that one previously prioritized/found interesting. https://doi.org/10.1093/bioinformatics/btt733

Overall, nice work. The paper was well-written. I hope the tool provides some help for people looking to explore novel junctions.

Is the rationale for developing the new method (or application) clearly explained?

Yes

Is the description of the method technically sound?

Yes

Are the conclusions about the method and its performance adequately supported by the findings presented in the article?

Yes

If any results are presented, are all the source data underlying the results available to ensure full reproducibility?

Yes

Are sufficient details provided to allow replication of the method development and its use by others?

Yes

Reviewer Expertise:

Computational biology, transcriptomics, human genetics

I confirm that I have read this submission and believe that I have an appropriate level of expertise to confirm that it is of an acceptable scientific standard.

## References

[1] : SplicePlot: a utility for visualizing splicing quantitative trait loci. *Bioinformatics* .2014;30(7) : 10.1093/bioinformatics/btt733 1025-6 10.1093/bioinformatics/btt733 24363378PMC3967110

# Peer review: Reviewer response for version 1

**DOI:** 10.5256/f1000research.78631.r118437

**Published:** 2022-01-05

**Authors:** George Wiggins

**Affiliations:** 1 Mackenzie Cancer Research Group, Department of Pathology and Biomedical Science, University of Otago, Christchurch, New Zealand

The authors have developed a novel method (Slinker) to visualize RNA isoforms that improves interpretation. The method requires condensing the genomic region to view only the sequence that is supported by RNA-sequencing reads.

 In my opinion Slinker does address the need in visualizing RNA isoforms that are assembled from RNA-sequencing data. A limitation that should be discussed is the reliance on StringTie2 for transcript assemble. RNA isoform discovery is a growing field, in which Slinker would offer great use, if adaptable to the development of new tools (e.g. FLAIR, bambu).

 Minor comments are listed below: 

- I have assumed long and short-read sequencing can be visualized equally? If not, this needs to be highlighted as a limitation in the discussion.
- In the discussion, the authors highlight how RNA species can help determine the pathogenicity of a given variants. For interpretation of RNA splicing events, it is important to determine whether splicing shift the coding frame. Is this possible to implement? This is not a requirement for indexing but a suggested improvement to Slinker. 

All figures are presented well but could be improved with increased font sizes. Additionally, is it possible to provide an HTML version of the one plot in the supplementary information or in the github repo?

- Figure3A has purple shading at the 5` end of the gene that I assume is not relevant, this should be removed for clarity.
- Please provide definitions for acronyms used in plots, e.g. superTranscript panels have TE, NE, TF etc.

Is the rationale for developing the new method (or application) clearly explained?

Yes

Is the description of the method technically sound?

Yes

Are the conclusions about the method and its performance adequately supported by the findings presented in the article?

Yes

If any results are presented, are all the source data underlying the results available to ensure full reproducibility?

Yes

Are sufficient details provided to allow replication of the method development and its use by others?

Yes

Reviewer Expertise:

Cancer genetics, RNA-sequencing, bioinformatics

I confirm that I have read this submission and believe that I have an appropriate level of expertise to confirm that it is of an acceptable scientific standard.
